# Rare copy number variants analysis identifies novel candidate genes in heterotaxy syndrome patients with congenital heart defects

**DOI:** 10.1186/s13073-018-0549-y

**Published:** 2018-05-30

**Authors:** Chunjie Liu, Ruixue Cao, Yuejuan Xu, Tingting Li, Fen Li, Sun Chen, Rang Xu, Kun Sun

**Affiliations:** 1 Department of Pediatric Cardiology, Xinhua Hospital, School of Medicine, Shanghai Jiao Tong University, Shanghai, China; 2 The Second Affiliated Hospital and Yuying Children’s Hospital of Wenzhou Medical University, Zhejiang, China; 3 Department of Cardiology, Shanghai Children’s Medical Center, School of Medicine, Shanghai Jiao Tong University, Shanghai, China; 4 Scientific Research Center, Xinhua Hospital, School of Medicine, Shanghai Jiao Tong University, Shanghai, China

**Keywords:** Copy number variants, Congenital heart defects, Heterotaxy, Zebrafish, Left-right

## Abstract

**Background:**

Heterotaxy (Htx) syndrome comprises a class of congenital disorders resulting from malformations in left-right body patterning. Approximately 90% of patients with heterotaxy have serious congenital heart diseases; as a result, the survival rate and outcomes of Htx patients are not satisfactory. However, the underlying etiology and mechanisms in the majority of Htx cases remain unknown. The aim of this study was to investigate the function of rare copy number variants (CNVs) in the pathogenesis of Htx.

**Methods:**

We collected 63 sporadic Htx patients with congenital heart defects and identified rare CNVs using an Affymetrix CytoScan HD microarray and real-time polymerase chain reaction. Potential candidate genes associated with the rare CNVs were selected by referring to previous literature related to left-right development. The expression patterns and function of candidate genes were further analyzed by whole mount in situ hybridization, morpholino knockdown, clustered regularly interspaced short palindromic repeats (CRISPR)/CRISPR-associated protein 9 (Cas9)-mediated mutation, and over-expressing methods with zebrafish models.

**Results:**

Nineteen rare CNVs were identified for the first time in patients with Htx. These CNVs include 5 heterozygous genic deletions, 4 internal genic duplications, and 10 complete duplications of at least one gene. Further analyses of the 19 rare CNVs identified six novel potential candidate genes (*NUMB*, *PACRG*, *TCTN2*, *DANH10*, *RNF115*, and *TTC40*) linked to left-right patterning. These candidate genes exhibited early expression patterns in zebrafish embryos. Functional testing revealed that downregulation and over-expression of five candidate genes (*numb*, *pacrg*, *tctn2*, *dnah10*, and *rnf115*) in zebrafish resulted in disruption of cardiac looping and abnormal expression of *lefty2* or *pitx2*, molecular markers of left-right patterning.

**Conclusions:**

Our findings show that Htx with congenital heart defects in some sporadic patients may be attributed to rare CNVs. Furthermore, *DNAH10* and *RNF115* are Htx candidate genes involved in left-right patterning which have not previously been reported in either humans or animals. Our results also advance understanding of the genetic components of Htx.

**Electronic supplementary material:**

The online version of this article (10.1186/s13073-018-0549-y) contains supplementary material, which is available to authorized users.

## Background

Heterotaxy (Htx) syndrome is a serious congenital malformation with high mortality and morbidity characterized by the failure to establish normal left-right (LR) body asymmetry. Patients often present with abnormal arrangement of the thoraco-abdominal organs, including ectopia of the heart, lungs, spleen, or liver [1]. The survival rate and outcomes of patients with Htx are unsatisfactory, as approximately 90% of cases are associated with complex congenital heart diseases, including malposition of the great arteries, presence of a single right ventricle, total anomalous pulmonary venous drainage, and double-outlet right ventricle [2, 3].

Researchers have made significant progress in enhancing our understanding of the molecular and cellular mechanisms that determine laterality during early embryogenesis. In the primitive node, asymmetry signaling is activated by leftward “nodal flow” created by the unidirectional rotation of monocilia. The asymmetry signals are then transmitted to the left lateral plate mesoderm, where they upregulate the expression of a series of left determinants, such as *Nodal*, left-right determination factor 2 (*Lefty2*), and paired-like homeodomain 2 (*Pitx2*). Several signaling pathways are involved in establishment of the LR axis, including Notch, Nodal, Hedgehog, Wnt, and transforming growth factor beta (TGF-β) [3–5]. In humans, mutations in several genes have been associated with Htx, including *CFC1*, *NODAL*, *ACVR2B*, *LEFTY2*, *GDF1*, *ZIC3*, *CRELD1*, and *NKX2.5* [6–13]. However, the mutations reported in these genes can explain only 10–20% of Htx cases; the underlying cause in the majority of patients remains unknown [14–16].

Copy number variants (CNVs) are DNA fragments whose copy number varies between individuals in a population due to duplication or deletion events. CNVs can range in size from 1 kilobase (kb) to several megabases (Mb). Numerous studies have demonstrated that a variety of diseases, especially syndrome-related diseases, are associated with CNVs. Several researchers recently reported a relationship between CNVs and Htx. Both Brueckner and Mills identified several novel rare CNVs in congenital heart disease patients with abnormal LR patterning [15–17], suggesting that CNVs may account for a proportion of patients. But the role of CNVs in the occurrence of Htx in patients with complex congenital heart disease should be examined in greater detail.

In this study, we identified 19 rare CNVs in 63 patients with Htx by genotyping their DNA using an Affymetrix CytoScan HD microarray. We further identified six potential candidate genes involved in several pathways reported to be related to LR development: ciliary proteome and function, Notch signaling pathway, or ubiquitination (ubiquitin ligase E3 family). Downregulation and over-expression of the Htx candidate genes *numb*, Parkin co-regulated gene (*pacrg*), tectonic family member 2 (*tctn2*), dynein axonemal heavy chain 10 (*dnah10*), and ring-finger protein 115 (*rnf115*) in zebrafish resulted in disruption of both heart looping and expression of *lefty2* or *pitx2*. To our knowledge, this study is the first to identify *DNAH10* and *RNF115* as novel Htx candidate genes. These two genes have not been previously implicated in LR patterning in either humans or animals; *numb*, *pacrg*, and *tctn2* have been linked to LR development in animals but are previously unreported in patients with Htx.

## Methods

### Patient ascertainment and study populations

Our study recruited patients with Htx in Xinhua Hospital and Shanghai Children’s Medical Center (SCMC) whose diagnoses were confirmed by echocardiography, cardiac catheterization examinations, computed tomography, abdominal ultrasonography, and other operation recordings. Patients exhibiting abnormal arrangement of the visceral organs and complex congenital heart disease were included, while those with complete situs solitus or other syndromes were excluded.

### Affymetrix CytoScan HD microarray analysis

Peripheral blood samples were obtained from each patient, and DNA was extracted using the QIAamp DNA Blood Midi Kit (Qiagen, Duesseldorf, Germany) following the manufacturer’s instructions. The CNVs were detected by CytoScan HD microarray platform (Affymetrix, Santa Clara, CA, USA), which is a high-density chip that contains 2,636,550 probes. In total, 59 samples passed initial quality control. Gains and losses were analyzed using Chromosome Analysis Suite (ChAS) software and the annotations of the Genome Reference Consortium (GRC) human reference genome version GRCh37 (hg19). The data were filtered, and only those regions larger than 50 kb comprising at least 25 contiguous markers were considered. Finally, we distinguished common CNVs from rare CNVs by comparing the results with the known CNVs in the Database of Genomic Variants (DGV, http://dgv.tcag.ca/) and Online Mendelian Inheritance in Man (OMIM, http://omim.org).

### Quantitative real-time polymerase chain reaction validation

The selected segments, which are related with Htx, were verified by quantitative real-time polymerase chain reaction (qPCR). The qPCR validation was performed according to SYBR® Premix Ex TaqTM II protocol (Applied TaKaRa). We used 50 ng/μL of genomic DNA in a 20 μL reaction, consisting of 10 μL of 2× SYBR Premix Ex Taq, 0.4 μL of 50× ROX Reference Dye II, 0.3 μL of forward primer, 0.3 μL of reverse primer, 8.0 μL of ddH_2_O, and 1 μL of DNA. Genomic DNAs extracted from healthy people were mixed, serving as normal controls, and the house-keeping gene GAPDH was used as the control in qPCR. The reactions were performed in triplicate.

### Whole-exome sequencing analysis and mutation detection

We performed whole-exome sequencing in patients with Htx and also in 100 healthy control people. The DNA was sequenced using the Illumina HiSeq 2500 platform at a commercial provider (Shanghai Biotechnology Co, Ltd., Shanghai, China). We defined functional mutations to be nonsynonymous mutations, stop-gain mutations, stop-loss mutations, frameshift or non-frameshift deletions or insertions, and splice site mutations.

### Zebrafish husbandry

Adult zebrafish (*Danio rerio*, AB line and Tg [cmlc2:EGFP] line) were raised under standard laboratory conditions with an automatic fish housing system (ESEN, Beijing, China) at 28 °C. All zebrafish experiments were conducted at the Institute of Neuroscience, Chinese Academy of Sciences, according to standard protocols. Embryo stages were determined according to their developmental morphology [18].

### Morpholino oligo injection and target gene knockdown

Morpholino oligos (MOs) designed against target genes were purchased from Gene Tools (Philomath, OR, USA) and dissolved in nuclease-free water. According to Gene Tools’ protocol, concentrations of MOs were checked by spectrophotometry (A265 in 0.1 N HCl). MOs were diluted to different working concentrations and 1 nL was pressure-injected into one-cell-stage embryos with a Picospritzer II injector.

The MOs for examining heart looping in morphants range from 2 ng to 8 ng in dosage: 8 ng *pacrg* MO, 8 ng *tctn2* MO, 8 ng *numb* MO, 8 ng *dnah10* MO, 2 ng *rnf115* MO, 8 ng *cfap46*/*ttc40* MO, and 8 ng *galnt11* MO per embryo. As negative controls, we injected 8 ng of standard control MO. A summary of MO doses and sequences is provided in Additional file 1: Table S1. The knockdown efficiencies of splice blocking and translation blocking MOs are illustrated in Additional file 2: Figure S1.

### CRISPR/Cas9-mediated mutation of *dnah10* and *rnf115* in zebrafish embryos

As previously reported, the clustered regularly interspaced short palindromic repeats (CRISPR)/CRISPR-associated protein 9 (Cas9) system was applied to introduce *dnah10* and *rnf115* gene mutation in zebrafish embryos [19]. The sequences of *dnah10* guide RNA (gRNA) (5′-GGCTCAGTTCTATGCTTACT -3′) and *rnf115* gRNA (5′-GGACAGTCTTGACTCTGAG -3′) were designed to target the sequences of mature *dnah10* and *rnf115*, respectively. We co-injected 600 pg zCas9 messenger RNA (mRNA) and 100 pg *dnah10* gRNA or 100 pg *rnf115* gRNA into zebrafish embryos at the one-cell stage. The gene mutation and knockout efficiency in F0 embryos was examined by PCR and sequencing analysis with the following primers:

*rnf115* forward: 5′-GAGAAGCACTGGTTCCGTCA-3′.

*rnf115* reverse: 5′-AACATACCCCTCAACAGCGG-3′.

*dnah10* forward: 5′-ATTCATCCAACGTGGAAACCA-3′.

*dnah10* reverse: 5′-GTCAGGACCTCGGTTTATTGTC-3′.

The knockout efficiency in F0 embryos of *dnah10* is 84.6%; for *rnf115* it is 75% (Additional file 2: Figure S2).

### mRNA synthesis and injection

Zebrafish total RNA was extracted from 1 to 2 days post fertilization (dpf) wild-type embryos (AB strain) and then was retrotranscribed into coding DNA (cDNA) with PrimeScript^tm^ RT reagent (TaKaRa, Shiga, Japan) according to the manufacturer’s instructions. The full-length coding sequence DNA of *tctn2*, *pacrg*, *galnt11*, *numb*, and *rnf115* was amplified using specific primers with restriction enzyme digestion loci, which were next subcloned into the pCS2+ vector (Additional file 1: Table S2). Positive clones selected by DNA sequencing were applied to generate the corresponding full-length mRNAs using a T7 or SP6 mMessage mMachine kit (Ambion). For the over-expression experiment, *pacrg*, *tctn2*, *numb*, and *galnt11* mRNAs were injected into one-cell-stage embryos and green fluorescent protein (GFP) was used as a negative control; for the rescue experiment, 6.25 pg *rnf115* mRNA was injected into one-cell-stage embryos mixed with 2 pg *rnf115* MO.

### Whole mount in situ hybridization

The whole mount in situ hybridization (WMISH) with anti-digoxigenin probes was performed according to the previously described protocol [20]. The anti-digoxigenin RNA probes (Roche) were synthesized with a length of 400–1300 necleotides, complementary to *numb*, *pacrg*, *tctn2*, *dnah10*, *rnf115*, *pitx2*, and *lefty2*, respectively (Additional file 1: Table S3).

The injected doses of MOs are from 2 ng to 8 ng for scoring of *pitx2* and *lefty2* expression in morphants: 8 ng *pacrg* MO, 8 ng *tctn2* MO, 8 ng *numb* MO, 8 ng *dnah10* MO, 2 ng *rnf115* MO, 8 ng *cfap46*/*ttc40* MO, and 4 ng *galnt11* MO per embryo. As negative controls, we injected 8 ng of standard control MO (Additional file 1: Table S1).

### Statistical analysis

In all figures, statistical comparisons between groups were analyzed by the chi-squared test (continuity corrected) or Fisher’s exact test. We defined *P* < 0.05 as statistically significant with *P < 0.05, **P < 0.01, ***P < 0.001.

## Results

### Clinical data

A total of 63 Chinese children with sporadic Htx were recruited. All of the patients exhibited abnormal arrangement of the visceral organs and complex congenital heart disease, not including complete situs solitus or other syndromes [21]. Among the patients we recruited, no one had central nervous system malformations, vertebral defects, or genitourinary malformations. According to the patients’ medical history, there was no family history of heterotaxy or other malformations. The patients’ ages ranged from 12 days to 113 months; 40 patients were male (63.5%) and 23 were female (36.5%). The detailed cardiac and extracardiac clinical phenotypes are summarized in Table 1. Pulmonary outflow obstruction was discovered in 56 patients, complete or partial atrioventricular canal in 20 patients, and single atrium or single ventricle in 35 patients. Twenty-seven patients had malposed or transposed great arteries, and 21 patients had double outlet of the right ventricle.

**Table 1:** Cardiac and extracardiac abnormalities in the patients with Htx

	Number of patients (%)
Sex	
Male	40 (63.5%)
Female	23 (36.5%)
Cardiac position	
Levocardia	15 (23.8%)
Dextrocardia	35 (55.6%)
Mesocardia	13 (20.6%)
Atrial arrangement	
Atrial situs inversus	20 (31.7%)
Isomerism of right atrial appendages	33 (52.4%)
Isomerism of left atrial appendages	7 (11.1%)
Ventricular arrangement	
Ventricular situs solitus	14 (22.2%)
Ventricular situs inversus	16 (25.4%)
Single ventricle (morphologic right)	23 (36.5%)
Single ventricle (morphologic left)	3 (4.8%)
Single ventricle (morphologic indeterminate)	5 (7.9%)
Other abnormal ventricle arrangement	2 (3.2%)
Bronchi	
Bilateral right bronchi (short)	34 (54.0%)
Bilateral left bronchi (long)	7 (11.1%)
Bronchial inversus	22 (34.9%)
Spleen	
Polysplenia	6 (9.5%)
Asplenia	29 (46.0%)
Single right spleen	21 (33.3%)
Single left spleen	7 (11.1%)
Stomach	
Right-sided stomach	38 (60.3%)
Left-sided stomach	19 (30.2%)
Stomach centrally situated	4 (6.3%)
Unknown	2 (3.2%)
Liver	
Left-sided liver	23 (36.5%)
Liver centrally situated	31 (49.2%)
Aortic arch	
Left aortic arch	17 (27.0%)
Right aortic arch	45 (71.4%)
Aortic arch centrally descending	1 (1.6%)
SVC	
Right SVC	8 (12.7%)
Left SVC	33 (52.4%)
Bilateral SVC	22 (34.9%)
IVC	
Interrupted IVC, hemiazygos vein continuation	1 (1.6%)
Interrupted IVC, azygos vein continuation	6 (9.5%)
Relationship of IVC and descending aorta	
IVC right of spine and descending aorta left of spine	4 (6.3%)
IVC left of spine and descending aorta right of spine	17 (27.0%)
IVC and descending aorta same side	32 (50.8%)
IVC left of spine and descending aorta anterior of spine	2 (3.2%)
IVC anterior of spine and descending aorta left of spine	1 (1.6%)

*SVC* superior vena cava, *IVC* inferior vena cava

### CNVs in patients with Htx and identification of candidate genes

To identify the molecular causes of Htx, an Affymetrix CytoScan HD microarray was used to identify possible pathogenic CNVs. A total of 59 samples passed initial quality control. Rare CNV segments were identified based on the following criteria: (1) CNV > 50 kb in size; (2) > 25 markers in each segment; (3) present at < 1‰ frequency or have < 50% overlap with published common CNVs or not found in the DGV (http://dgv.tcag.ca/); (4) not identified in either a dataset with microarray results of 216 normal Chinese individuals or another dataset of 720 Chinese non-heterotaxy patients with developmental delay/intellectual disability (DD/ID) (Additional file 1: Table S4). Finally, we identified 19 rare CNVs in 14 patients with Htx (Table 2). The percentage of subjects with rare CNVs was 23.7% (14 of 59 Htx subjects). The selected CNVs ranged in size from 57 to 1009 kb. These CNVs included 5 heterozygous genic deletions, 4 internal genic duplications, and 10 complete duplications of at least one gene [15].

**Table 2:** Nineteen rare copy number variants identified in patients with heterotaxy

ID	Chromosome	Genomic coordinates	Type	Size (kbp)	Genes altered
5	4q24	104,554,264–105,123,728	Internal dup	569.464	*TACR3*
5	6p22.2	26,019,198–26,227,973	Genic dup	208.775	*HIST1H3A, HIST1H4A, HIST1H4B, HIST1H3B, HIST1H2AB, HIST1H2BB, HIST1H3C, HIST1H1C, HFE, HIST1H4C, HIST1H1T, HIST1H2BC, HIST1H2AC, HIST1H1E, HIST1H2BD, HIST1H2BE, HIST1H4D, HIST1H3D, HIST1H2AD, HIST1H2BF, HIST1H4E, HIST1H2BG, HIST1H2AE, HIST1H3E*
7	1q21.1	145,625,128–145,927,662	Genic del	302.534	***RNF115*** *, CD160, PDZK1, GPR89A, GPR89C, PDZK1P1*
10	5q23.1	115,247,380–115,683,172	Genic dup	435.792	*AP3S1, AQPEP, LOC644100, COMMD10*
16	6p12.1	54,138,106–54,277,341	Genic dup	139.235	*TINAG*
18	12p13.33	173,786–356,461	Genic dup	182.675	*IQSEC3, LOC574538, SLC6A12, SLC6A13*
20	12q24.31	123,357,010–124,310,519	Genic dup	953.509	*VPS37B, ABCB9, OGFOD2, ARL6IP4, PITPNM2, MIR4304, LOC100507091, MPHOSPH9, C12orf65, CDK2AP1, SBNO1, SETD8, RILPL2, SNRNP35, RILPL1, MIR3908, TMED2, DDX55, EIF2B1, GTF2H3,* ***TCTN2*** *, ATP6V0A2,* ***DNAH10***
20	19q13.32	47,308,130–47,418,258	Genic dup	110.128	*SNAR-E, AP2S1*
26	11q12.2	60,408,411–60,465,698	Genic del	57.287	*LINC00301*
31	10p15.1	6,254,055–6,374,584	Internal dup	120.529	*PFKFB3, LOC399715*
34	4q22.2	93,875,432–93,988,049	Genic del	112.617	*GRID2*
39	8q11.1q11.21	47,398,661–48,407,568	Genic dup	1008.907	*LINC00293, LOC100287846, KIAA0146*
40	14q24.2	73,620,299–73,786,493	Genic dup	166.194	*PSEN1, PAPLN,* ***NUMB***
43	4q24	101,476,709–101,668,938	Genic del	192.229	*EMCN-IT3*
59	2q24.1	157,170,397–157,315,649	Internal dup	145.252	*NR4A2, GPD2*
59	6q26	163,549,870–163,842,358	Genic dup	292.488	***PACRG*** *, PACRG-AS1, DKFZp451B082, CAHM, QKI*
59	9p22.2	16,826,417–16,931,236	Internal dup	104.819	*BNC2*
63	3q25.32	158,198,274–158,256,949	Genic del	58.675	*RSRC1*
63	10q26.3	134,358,785–134,921,135	Genic dup	562.35	*INPP5A, NKX6–2,* ***TTC40*** *, LOC399829, GPR123*

Bold items are candidate genes we identified from rare CNVs

*Genic del* deletion of at least one coding exon, *Genic dup* full duplication of at least one gene, *Internal dup* duplication of internal exons

Among the 19 rare CNVs, none of the common known Htx-related genes, including *ZIC3*, *CFC1*, *NKX2.5*, *GDF1*, *NODAL*, *LEFTY1*, *LEFTY2*, *ACVR2B*, *DANH5*, *DNAH11*, *DNAI1*, *FOXH1*, *CRELD1*, or *GALNT11*, were identified. In order to correlate the phenotypes of the Htx syndrome patients to specific pathologic genes, we first examined the function of the genes associated with the 19 rare CNV segments (Additional file 1: Table S5). Candidate genes were identified based on the following criteria: (1) participation in ciliary proteome and function; (2) relation to signaling pathways Notch, Nodal, Hedgehog, Wnt, and TGF-β; (3) member of ubiquitin ligase E3 family. E3 ubiquitin ligases were reported to play important roles in the Nodal signaling pathway, cilia formation, and cilia assembly [22–24]. We finally found six potential candidate genes in five CNV segments in five subjects: *NUMB* [MIM: 603728], *PACRG* [MIM: 608427], *TCTN2* [MIM: 613846], *DANH10* [MIM: 605884], *RNF115*, and cilia and flagella associated protein 46 [*CFAP46/TTC40*].

**Fig. 1:**
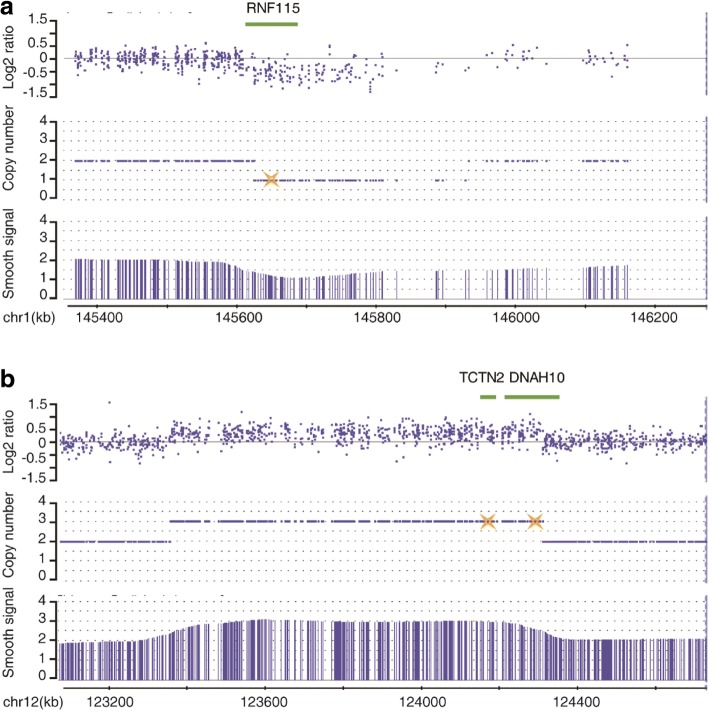
Chromosomal view of rare CNVs in candidate Htx patients and the verified results of qPCR. **a** CytoScan HD array presents a 302.5-kb deletion of 1q21.1 involving *RNF115*. **b** A 953.5-kb duplication at 12q24.31 affecting both *TCTN2* and *DNAH10*. In data (**a**, **b**), the *upper panel* depicts log2 ratio data, the *middle panel* depicts the copy number duplications or deletions, and the *lower panel* depicts smooth signals of indicated segments. Locations of genes implicated in Htx are shown in the *top*. Results of qPCR are denoted by *yellow stars*

Verification by qPCR showed that these five rare CNVs comprise four duplications and one deletion (Fig. 1 and Additional file 2: Figure S3). The qPCR results and clinical diagnosis of the five patients are summarized in Table 3.

**Table 3:** Clinical phenotypes of heterotaxy patients with CNVs carrying candidate genes

ID	Segments	Sizes (kb)	Copy numbers	Genes	Patients’ cardiac abnormalities	Extracardiac abnormalities
7	1q21.1 (145,625,128–145,927,662)	302.534	1 del	*RNF115*	D, SA, PAVC, LSVC	RAA, BI, right spleen, RSS, LSL
20	12q24.31 (123,357,010–124,310,519)	953.509	3 dup	*TCTN2, DNAH10*	D, SA, TA, TGA, PA, VSD, PDA, LSVC	RAA, BI, right spleen, RSS, LSL
40	14q24.2 (73,620,299–73,786,493)	166.194	3 dup	*NUMB*	D, DORV, PS, VSD, LSVC, SIV	RAA, BI, right spleen, RSS, LSL
59	6q26 (163,549,870–163,842,358)	292.488	3 dup	*PACRG*	D, SA, SV, MGA, PS, CAVC, LSVC, IRAA	LAA, BRB, asplenia, LSS, LCS
63	10q26.3 (134,358,785–134,921,135)	562.35	4 dup	TTC40	D, DORV, PS, VSD, PDA, LSVC, SIV	RAA, BI, right spleen, RSS, LSL

*dup* duplication, *del* deletion, *PDA* patent ductus arteriosus, *PS* pulmonary stenosis, *VSD* ventricle septum defect, *CAVC* complete atrioventricular canal, *PAVC* partial atrioventricular canal, *D* dextrocardia, *SA* single atrium, *SV* single ventricle, *TA* tricuspid atresia, *PA* pulmonary atresia, *TGA/MGA* translocation of great arteries/malposition of great arteries, *DORV* double outlet right ventricle, *IRAA* isomerism of right atrial appendages, *SIV* superior-inferior ventricle, *LSVC* left superior vena cava, *RAA* right aortic arch, *LAA* left aortic arch, *BI* bronchial inversus, *BRB* bilateral right bronchi (short), *RSS* right-sided stomach, *LSS* left-sided stomach, *LSL* left-sided liver, *LCS* liver centrally situated

To determine whether the patients identified as carrying CNVs of the candidate genes had mutations in other known laterality-related genes (e.g., *ZIC3*, *CFC1*, *NKX2.5*, *GDF1*, *NODAL*, *LEFTY1*, *LEFTY2*, *ACVR2B*, *DANH5*, *DNAH11*, *DNAI1*, *FOXH1*, *CRELD1*, and *GALNT11*), we screened the coding sequences of these genes using whole-exome sequencing analysis. Aside from a nonsynonymous heterozygous mutation (c. 841A > G, p.Trp281Arg) in *LEFTY1* (Additional file 2: Figure S4 and Additional file 1: Table S6) in one patient with a CNV of *CFAP46*, the results revealed no functional mutations in these laterality-related genes.

### Expression patterns of candidate genes in zebrafish

**Fig. 2:**
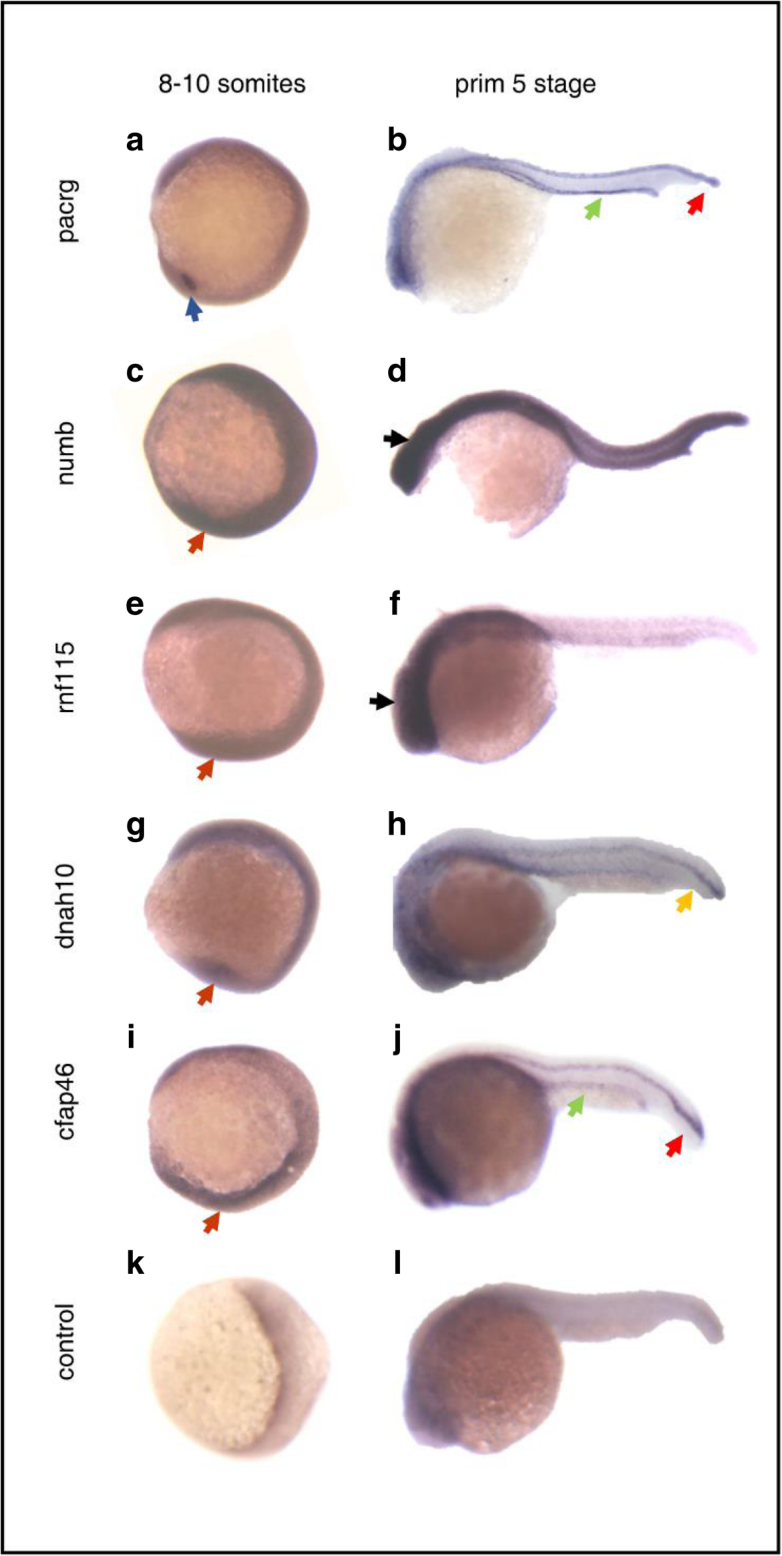
Whole mount in situ hybridization analysis of candidate genes at two stages: 8–10 somites and primordium 5 stage. **a**, **c**, **e**, **g**, **i**, **k** Results of in situ hybridization of candidate genes and standard control at 13–15 hpf (8–10 somites). Embryos are viewed laterally with anterior to the top to examine KV expression. **b**, **d**, **f**, **h**, **j**, **l** Results of in situ hybridization of candidate genes and standard control at 24 hpf (primordium 5 stage). Lateral view of embryos with anterior to the *left*. KV (*blue arrow*), floor plate (*red arrows*), pronephric duct (*green arrows*), notochord (*yellow arrow*), head (black arrows), ubiquitous expression (*orange arrows*)

Zebrafish were used as a model organism to further elucidate the roles of the candidate genes in regulating organ laterality, as all six of the candidate genes have orthologs in zebrafish. We examined the gene expression patterns at two developmental stages using WMISH with digoxigenin-labeled RNA as probes: the 8–10 somite stage (13–15 h post fertilization [hpf]; symmetry was first broken at Kupffer’s vesicle [KV]) and the primordium 5 stage (24 hpf; heart begins to beat). WMISH analysis of the *tctn2* gene was unsuccessful. The *pacrg* gene was expressed in the KV in the 8–10 somite stage, and was expressed in the pronephric duct and floor plate in the primordium 5 stage (Fig. 2a, b). In the 8–10 somite stage, *numb*, *rnf115*, *dnah10*, and *cfap46* exhibited nearly ubiquitous expression patterns (Fig. 2c, e, g, i). In the primordium 5 stage, however, these four genes exhibited more localized expression patterns: *numb* and *rnf115* were both expressed in the brain (Fig. 2d, f); *dnah10* was restricted to the notochord (Fig. 2h); *cfap46* was expressed in the pronephric duct and floor plate (Fig. 2j).

### Knockdown and mutation of the candidate genes disturbs cardiac looping

**Fig. 3:**
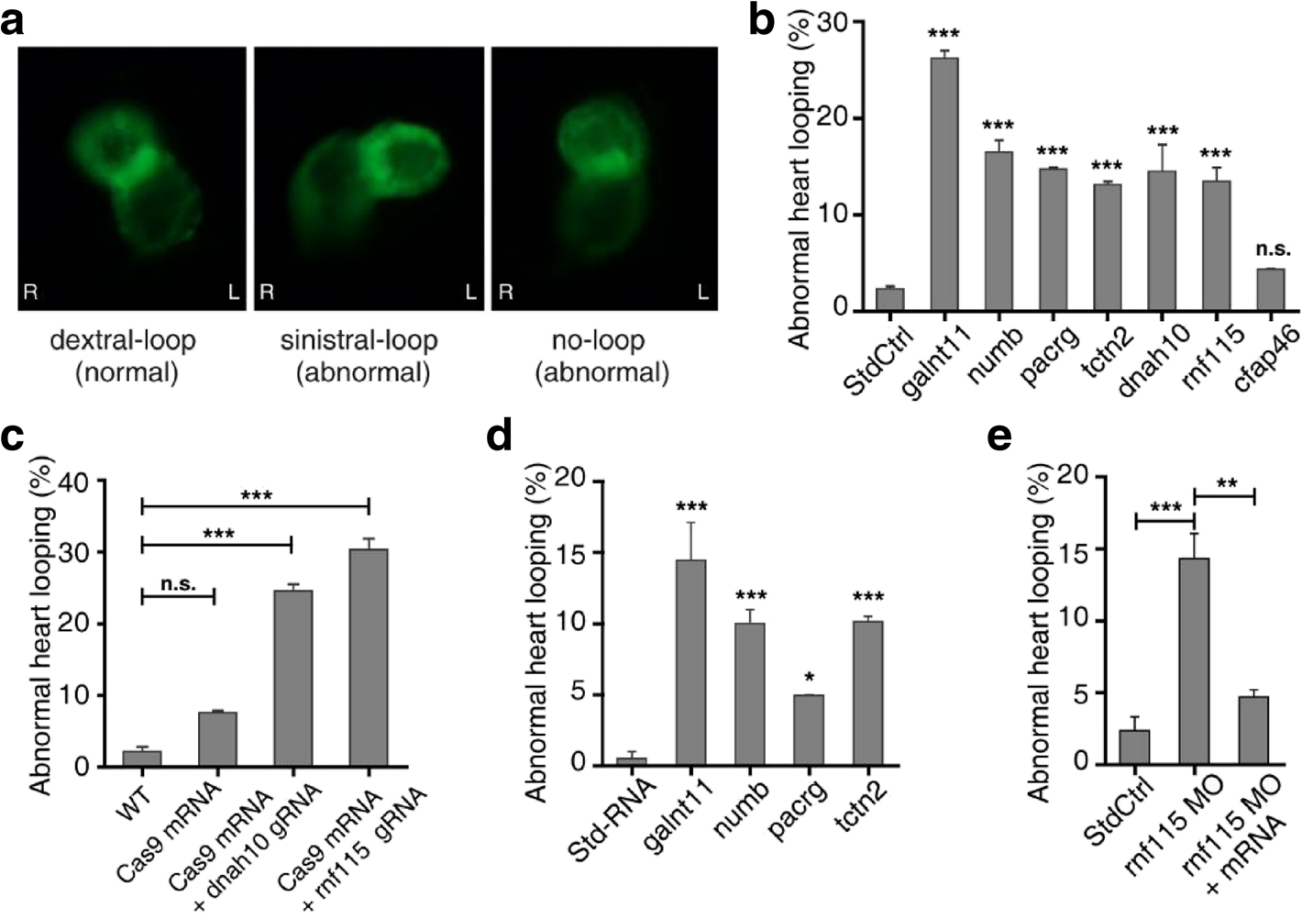
Loss of function of candidate genes in zebrafish disturbed cardiac looping. **a** Zebrafish heart shows normal dextral loop, abnormal sinistral loop, and no-loop types in cmlc2:eGFP morphants in ventral view. **b** The percentage of abnormal heart looping with MO injected. The experiments were repeated 3 times, and at each time > 70 embryos were examined for each group. **c** Summary of the abnormal heart looping of *dnah10* and *rnf115* mutations generated by co-injection of zebrafish Cas9 mRNA 600 pg and *dnah10* gRNA 100 pg or *rnf115* gRNA 100 pg. The experiments were repeated 3 times, and at each time > 71 embryos were examined for each group. **d** Percentage of embryos that exhibit abnormal cardiac looping with mRNA over-expressed. **e** The *rnf115* mRNA can rescue LR randomization. The abnormal heart looping phenotype which is induced by *rnf115* MO can be rescued by 6.25 pg *rnf115* mRNA. Heart looping direction was assayed in zebrafish at stage 2 dpf. *Bars* show the total percent of abnormally looped heart including two types: no-loop and sinistral loop heart. Standard control MO (*StdCtrl*) is negative control. *galnt11* is used as positive control. Error bars represent the standard error of the mean (SEM). *P < 0.05, **P < 0.01, ***P < 0.001, respectively vs. StdCtrl. *WT*, wild type

Expression of the candidate genes in zebrafish was downregulated using MO knockdown to examine the effect on cardiac looping, which depends on normal LR patterning. Three heart tube morphologies occur in zebrafish: dextral loop (d-loop), sinistral loop (s-loop), or no loop (Fig. 3a) [25]. In our study, *galnt11* was used as a positive control. *GALNT11*, encoding the polypeptide N-acetylgalactosaminyltransferase 11, was previously identified as a candidate Htx gene in a patient by CNV analysis. The gene plays an important role in the development of the LR axis by activating the Notch signaling pathway and modulating the balance between motile and nonmotile cilia [15, 26]. A standard control provided by Gene Tools was injected as a negative control.

Zebrafish embryos were injected with MOs at the one-cell stage, and the direction of heart looping was assessed at 48 hpf. We found that MO knockdown of five of the six candidate genes led to altered cardiac looping phenotypes. Compared with the negative control (2.35% abnormality), the phenotypes of five morphants (*numb*, *pacrg*, *tctn2, dnah10, and rnf115*) differed significantly, with 13.16–26.2% of embryos exhibiting either an L-loop or no loop (*P* < 0.001). However, knockdown of *cfap46* had no significant effect on the direction of heart looping (*P* > 0.05) (Fig. 3b).

Among the five candidate genes, *DNAH10* and *RNF115* were found to be novel candidate Htx genes in both humans and animals. *dnah10* and *rnf115* mutations induced by co-injecting zebrafish codon-optimized Cas9 mRNA and *dnah10* or *rnf115* gRNA also led to a significant increase of abnormal heart looping in F0 embryos (Fig. 3c; *P* < 0.001), which demonstrates the roles that *dnah10* and *rnf115* play in LR patterning.

### Over-expression of the candidate genes disturbs cardiac looping

The five candidate genes with phenotypes in MO-knockdown embryos were then divided into two groups based on the clinical copy number in the Htx patients: four duplicated genes (*numb*, *pacrg*, *tctn2*, *dnah10*), and one deleted gene (*rnf115*). The functions of the duplicated genes were examined using mRNA over-expression analyses. Rescue of the deleted gene, *rnf115*, was conducted using an *rnf115-*pCS2^+^ plasmid.

Compared with negative control (1% abnormality), over-expression of the candidate genes had a significant effect on cardiac looping: *numb* (10.0% abnormality, *P* < 0.001); *tctn2* (10.2% abnormality, *P* < 0.001); *pacrg* (4.95% abnormality, *P* < 0.05) (Fig. 3d). Over-expression of the duplicated gene *dnah10* could not be carried out because its mRNA was too long (14,062 bases). Remarkably, *rnf115* MO knockdown led to 14.3% of abnormal cardiac looping, and injection of zebrafish with *rnf115* mRNA rescued the normal phenotype (4.7% abnormality). This shows that this gene has a specific function in LR patterning (Fig. 3e).

### Candidate genes exhibit global effects on early signaling pathways in LR development

**Fig. 4:**
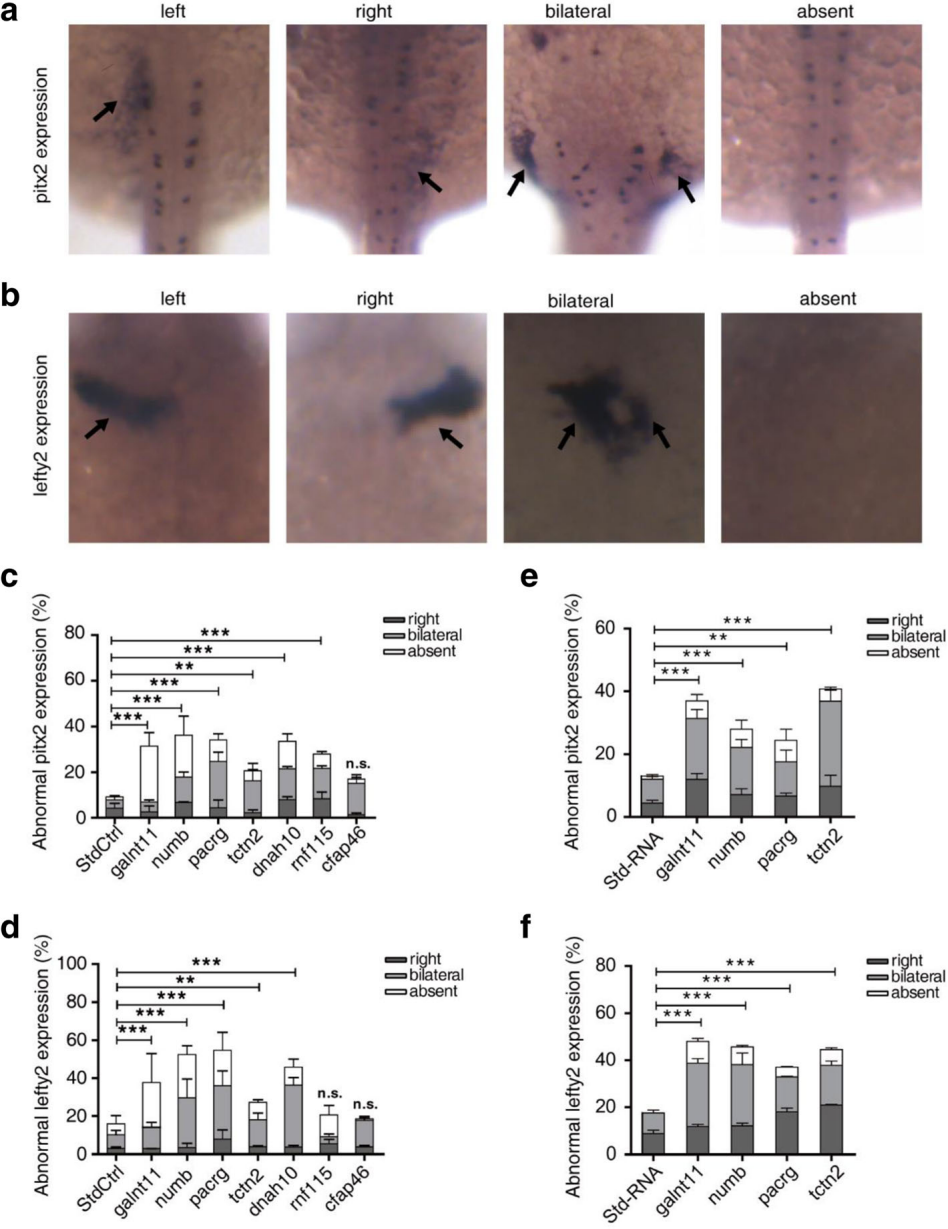
Analysis of *pitx2* and *lefty2* expression patterns in the lateral plate mesoderm in 18–22 somites. **a** The expression of *pitx2* exhibits four patterns, left, right, bilateral, or absent, in the posterior lateral plate mesoderm of zebrafish embryos. **b** Morphants show left, right, bilateral or absent *lefty2* expression in the cardiac field. **c**, **d** Summary of abnormal *pitx2* and *lefty2* mRNA expression in zebrafish morphants. **e**, **f** Summary of abnormal *pitx2* and *lefty2* mRNA expression in zebrafish with mRNA over-expressed. Embryos are viewed dorsally with anterior *to the top*. *Bars* show the percent of abnormal *pitx2* and *lefty2* expression including three types: right, bilateral, absent expression. Standard control (*StdCtrl* or *Std-RNA*) is negative control. *galnt11* is used as positive control. Error bars represent the SEM. *P < 0.05, **P < 0.01, ***P < 0.001, respectively, vs. standard control

An abnormal cardiac looping pattern can result from either disruption of early common laterality pathways or specific heart field effects. Based on the early expression patterns of the candidate genes and the patients’ clinical information of more than one organ malposition, we hypothesized that the abnormal cardiac looping phenotype results from disruption of early signaling pathways during LR development. To test this hypothesis, we examined the expression patterns in zebrafish morphants of *pitx2* and *lefty2*, which are markers of the early common laterality pathway in the 18–22 somite stage. The *pitx2* gene encodes a transcription factor that relays LR-patterning information necessary for proper organogenesis, whereas *lefty2* encodes a protein of the TGF-β superfamily that inhibits nodal activation. The embryonic expression of *pitx2* and *lefty2* exhibited both normal (left side) and abnormal (right side, bilateral, absent) patterns (Fig. 4a, b). Negative control morphants exhibited approximately 9.1% abnormal *pitx2* expression and 16.0% abnormal *lefty2* expression. Morphants injected with *galnt11* positive control exhibited 31.4% of *pitx2* and 37.7% of *lefty2* abnormality (*P* < 0.001). The *numb*, *pacrg*, *tctn2*, and *dnah10* morphants exhibited significant abnormal *pitx2* and *lefty2* expression patterns (20.6–36.2 of *pitx2* abnormality and 27.3–54.7% of *lefty2* abnormality; *P* < 0.001). The *rnf115* morphants showed 28.0% abnormality in *pitx2* expression (*P* < 0.001) but did not exhibit significant abnormal *lefty2* expression. Consistent with the results above, *cfap46* morphants exhibited no significant abnormalities in either *pitx2* or *lefty2* expression (Fig. 4c, d).

Then, we analyzed the expression of *pitx2* and *lefty2* in embryos with over-expression of genes found in genic duplications (*numb*, *pacrg*, *tctn2*). The results showed that the embryonic expression of *pitx2* and *lefty2* also exhibited both normal (left side) and abnormal (right side, bilateral, absent) patterns. Negative control exhibited approximately 13.0% abnormal *pitx2* expression and 17.7% abnormal *lefty2* expression. Over-expression of *galnt11* positive control exhibited 36.8% of *pitx2* and 47.8% of *lefty2* abnormality (*P* < 0.001). Over-expression of *numb*, *pacrg*, and *tctn2* exhibited significant abnormal *pitx2* and *lefty2* expression patterns (24.4–40.1% of *pitx2* abnormality and 36.9–46.5% of *lefty2* abnormality; *P* < 0.01) (Fig. 4e, f).

### Whole-exome sequencing and mutation screening

In order to further explore the relationship between the candidate genes and Htx, we screened the sequencing data of the five candidate genes for rare mutations in 65 Htx patients without mutations of other known laterality-related genes and rare CNVs. Filtering criteria were set as follows: (1) variants are located in exonic or splicing region, (2) exclude synonymous variants, (3) frequency is lower than 0.1% according to public variant databases 1000 Genomes and Exome Aggregation Consortium (ExAC), (4) exclude variants detected in our 100 normal Chinese individuals or 2000 non-heterotaxy patients, and (5) at least one scoring software analysis suggests that mutation is deleterious. Finally, six rare heterozygous mutations in *DNAH10*, *RNF115*, *TCTN2*, and *NUMB* were detected in six sporadic Htx patients (Additional file 2: Figure S5 and Additional file 1: Table S7).

## Discussion

Htx comprises a class of congenital disorders resulting from malformations in LR body patterning, but the underlying cause in the majority of patients remains unknown. In an effort to elucidate the molecular mechanism underlying the pathogenesis of Htx, we recruited 63 children with Htx but free of other syndromes. CNV analyses identified 19 rare CNVs. Further analyses revealed that six candidate genes associated with the 19 rare CNV segments were related with pathways reported to be involved in the regulation of LR development. Downregulation and over-expression of the candidate genes in zebrafish demonstrated that five genes (*numb*, *pacrg*, *tcnt2*, *dnah10*, and *rnf115*) strongly affect morphologic cardiac looping as well as the pattern of *pitx2* and *lefty2* expression. Moreover, we detected rare mutations in the coding sequence of candidate genes *DNAH10*, *RNF115*, *TCTN2*, and *NUMB* in Htx patients.

The study demonstrated that rare CNVs play an important role in the pathogenesis of Htx in patients. CytoScan HD microarray is a good method for copy-number analyses. However, balanced chromosomal rearrangements such as inversions or balanced translocations could be potentially missed on the CytoScan HD microarray. In our study, the percentage of subjects with rare CNVs (23.7%, 14 of 59 Htx subjects) was higher than that previously reported [15, 27]. The size of these rare CNVs varied from 57 to 1009 kb. One duplication segment at 10q26.3 (genomic coordinates 134,358,785–134,921,135, involving only five genes) identified in our study was included in previously published deletion segments associated with Htx: a terminal CNV beginning at 10q26.13 [15, 17]. The remaining 18 segments were never reported.

Among the five candidate genes we identified, the *numb*, *pacrg*, and *tctn2* genes had been linked to LR development in animals but not in humans. The gene *NUMB* encodes an endocytic adaptor protein that plays a role in the determination of cell fate. Consistent with previous reports, zebrafish *numb* was expressed ubiquitously in the early developmental stage in the present study. Over-expression of *numb* has been shown to suppress Notch activity, thus causing bilateral distribution of *lefty2* expression and disturbed heart tube looping [28]. This observation strongly supports our data showing that not only over-expression but also downregulation of *numb* in zebrafish leads to abnormal cardiac looping and a random distribution of *lefty2* and *pitx2* expression.

The genes *pacrg* and *tctn2* are previously reported to regulate ciliary function [29–31]. Cilia play a pivotal role in earlier LR development. The gene *pacrg* is locally expressed in the KV in zebrafish, where LR asymmetry is initially established. *Xenopus* embryos injected with *pacrg* MO exhibit LR laterality defects as well as gastrulation and neural tube closure defects [29, 31]. *TCTN2* encodes a type I membrane protein of the tectonic family. *TCTN2* was linked to Joubert syndrome, a ciliopathy disease. *Tctn2* knockout mice exhibit ventricular septal defects and a right-sided stomach, suggesting that *Tctn2* plays a role in laterality defects [30]. Our data further show that over-expression of *pacrg* or *tctn2* in zebrafish also leads to laterality defects. Moreover, this is the first report identifying CNVs of *NUMB*, *PACRG*, and *TCTN2* in patients with Htx.

To date, no reports have linked *DNAH10* and *RNF115* with LR patterning in either humans or animals. The gene *dnah10,* expressed in cilia and flagella, is a component of the inner dynein arms, which are attached to the peripheral microtubule doublets; as a protein involved in ATP production, DNAH10 participates in protozoan flagellar motility [32, 33]. Recent studies have demonstrated that mutations in other cilia dynein heavy-chain genes (such as *DNAH5*, *DNAH9*, and *DNAH11*) can cause primary ciliary dyskinesia (PCD). PCD is a severe inherited disorder that results from defects in flagellar and ciliary axoneme substructures and is characterized by male infertility, respiratory diseases, and LR laterality in 50% of affected individuals [34–36]. We first identified the CNVs of *DNAH10* in patients with Htx, and found that knockdown and mutation of *dnah10* in zebrafish disturb the LR development and *pitx2* and *lefty2* expression patterns. Moreover, we found that *dnah10* mRNA is highly expressed in the caudal notochord in the primordium 5 stage. The notochord breaks bilateral symmetry by altering cell shape and cilia distribution [37]. *DNAH10* may regulate LR patterning by affecting the notochord, which could alter the function of cilia. In addition, both *TCTN2* and *DNAH10*, located within the same CNV segment, can affect the development of LR patterning, and whether there is potential interaction between them needs further research.

The only deleted gene identified in our study, *RNF115*, also known as breast cancer-associated gene 2 (*BCA2*), is a type of E3 ubiquitin ligase. E3 ubiquitin ligases play important roles in auto-ubiquitination activity, depending on their RING domain. *RNF115* mRNA is expressed at moderate levels in the heart, skeletal muscle, and testis [38]. Previous findings indicated that ubiquitination by E3 ligases regulates a diverse array of cellular functions, such as cilia formation and assembly, and LR development-related signaling pathways (e.g., Nodal signaling) [22–24]. However, the relationship between *RNF115* and LR asymmetry remains unknown. In our study, *rnf115* morphant and mutant zebrafish exhibited disturbed cardiac looping, and *rnf115* mRNA rescued the normal phenotype. Moreover, *rnf115* morphants exhibited random distribution of *pitx2* expression but minimal perturbations in the *lefty2* expression pattern. According to the previously reported pathogenesis of Htx, asymmetric Nodal signaling activates *pitx2* in the left lateral plate mesoderm via the Smad-FoxH1 pathway [4]. Our data suggest that *rnf115* may function in the ubiquitination of Nodal-downstream genes (the genes in the Smad-FoxH1 pathway) or directly act on *pitx2*, to regulate LR patterning, which cannot alter the expression of *lefty2*. However, the specific mechanism through which *DNAH10* and *RNF115* direct LR axis development remains to be further investigated.

*TTC40* encodes cilia and flagella associated protein 46, which is reported to play a role in the occurrence of nasopharyngeal carcinoma and acute myeloid leukemia [39, 40]. In our study, zebrafish with knockdown of this gene did not exhibit an abnormal phenotype in terms of LR patterning. Moreover, in the patient in our study with a CNV of *CFAP46*, Htx might have been caused by a nonsynonymous mutation (c. 841 A > G, p.Trp281Arg) in *LEFTY1* instead.

## Conclusions

Our results demonstrate that Htx in some sporadic patients may be attributed to rare CNVs. Moreover, we identified candidate genes in several novel segments of rare CNVs, some of which have never before been reported as related to LR patterning. Downregulation or over-expression of the candidate genes in zebrafish disturbed the development of LR asymmetry. We believe the results of our study advance the understanding of Htx and will aid in its diagnosis. However, according to previous literature and the results of our study [41, 42], we do not have sufficient evidence to show that the genes reported in this paper are causal for heterotaxy. They are only candidate genes for Htx. This is the limitation of our study. The five genes identified in our paper are reported for the first time in a heterotaxy population, and our study can be used as the first evidence for the future research of the pathogenic genes of Htx. This is hoped to be further confirmed in other populations. In addition, other rare CNVs that we did not study might also play significant roles in development of LR asymmetry. Two novel candidate genes identified in the present study, *DANH10* and *RNF115*, should be examined in greater detail with respect to their role in the pathogenesis of defects in LR patterning.

## Additional files

Additional file 1: **Table S1.** MO sequences, injection doses, and total embryo numbers analyzed for heart looping and gene expression. **Table S2.** Specific primers and vector used to produce genes’ full-length mRNA. **Table S3.** Antisense RNA probes conducted for whole mount in situ hybridization. **Table S4.** The frequency of each candidate CNV in normal Chinese individuals and non-heterotaxy patients with developmental delay/intellectual disability. **Table S5.** The function of the genes associated with the 19 rare CNV segments. **Table S6.** The bioinformatics information on the variant of *LEFTY1* in the patient with CNV of *TTC40* (*CFAP46*). **Table S7.** The bioinformatics information on the variants of candidate genes. (PDF 369 kb)

Additional file 2: **Figure S1.** Knockdown efficiency of splice blocking and translation blocking MOs in zebrafish embryos. **Figure S2.** The knockout efficiency of *dnah10* and *rnf115* by CRISPR/Cas9. **Figure S3.** Chromosomal view of rare CNVs in candidate Htx patients and the verified results of qPCR. **Figure S4.** Gene sequencing peak shows a nonsynonymous heterozygous mutation in *LEFTY1* in the patient with CNV of *TTC40* (*CFAP46*). **Figure S5.** Rare variations were detected in Htx patients. (PDF 792 kb)

## Abbreviations

CNV: Copy number variant
d-loop: Dextral loop
gRNA: Guide RNA
hpf: Hours post fertilization
Htx: Heterotaxy
kb: Kilobase
KV: Kupffer’s vesicle
LR: Left-right
Mb: Megabase
MO: Morpholino oligo
PCD: Primary ciliary dyskinesia
qPCR: Quantitative real-time polymerase chain reaction
s-loop: Sinistral loop
WMISH: Whole mount in situ hybridization
