# Supplementary material for: Rare copy number variants analysis identifies novel candidate genes in heterotaxy syndrome patients with congenital heart defects
Source: Genome Med. 2018 May 30;10:40. doi: 10.1186/s13073-018-0549-y (PMC5975672; doi:10.1186/s13073-018-0549-y)
Supplement: Supplementary file 1 — Table S1. MO sequences, injection doses, and total embryo numbers analyzed for heart looping and gene expression. Table S2. Specific primers and vector used to produce genes’ full-length mRNA. Table S3. Antisense RNA probes conducted for whole mount in situ hybridization. Table S4. The frequency of each candidate CNV in normal Chinese individuals and non-heterotaxy patients with developmental delay/intellectual disability. Table S5. The function of the genes associated with the 19 rare CNV segments. Table S6. The bioinformatics information on the variant of LEFTY1 in the patient with CNV of TTC40 (CFAP46). Table S7. The bioinformatics information on the variants of candidate genes. (PDF 369 kb) [file 13073_2018_549_MOESM1_ESM.pdf]

Additional file 1：Supplemental Tables S1-S7

Additional file 1: Table S1. MO sequences, injection doses, and total embryo numbers analyzed for heart looping and gene expression

Gene	MO sequence 5' 3'	MO's function	Blocking site	Knockdown efficacy analysis	Dose (ng) for heart looping analysis	Numbers for heart looping analysis	Dose (ng) for gene expression analysis	Numbers for pitx2 expression analysis	Numbers for lefty2 expression analysis
numb	GGAAACTCTGCCGTAGCTTATTCAT	translation blocking	ATG	western blot	8	258	8	134	128
pacrg	TAGCCAAAGGTTCAAAGGTTCTCAT	translation blocking	ATG	western blot	8	333	8	138	120
tctn2	TTCGCTCAAAGTGGACTTACCAACA	splice blocking	E1I1	western blot	8	235	8	136	157
dnah10	AGATGAGAATCACGCACATGGTTGA	splice blocking	E5I5	reverse transcription PCR	8	285	8	161	155
rnf115	CTACCAGCGACACGGACTCACCG	splice blocking	E1I1	reverse transcription PCR	2	230	2	168	152
cfap46/ ttc40	CTTAGTGAACAGTGTTTTGACGTAC	splice blocking	E1I1	reverse transcription PCR	8	227	8	134	174
galnt11	GCCATTATCAGGCCCTCGCTGATGA	translation blocking	ATG	western blot	8	253	4	97	139
Standard control	CCTCTTACCTCAGTTACAATTTATA	—	/	—	8	337	8	158	161

Additional file 1: Table S2. Specific primers and vector used to produce genes' whole-length mRNA

Gene	Primer F	Primer R	Length (bp)	Restriction Enzyme Digestion	Vector
galnt11	CGGGATCCTCACCAGCGAGTAACGG	CCGCTCGAGCGTCAGACCAAGGCTTCA	2060	BamHI, XhoI	pSC2+
pacrg	CGGGATCCGAGTTTTGTTTCTGTTTATCGG	CCGCTCGAGCAGAAATAAGAAGTGTAACATAAGGT	853	BamHI, XhoI	pSC2+
numb	AGCGAATTCATGAATAAGCTG	TCCCTCGAGTTATAGTTCAATCTCAAA	1857	BamHI, XhoI	pSC2+
tctn2	CGGAATTCTCCAGGCGAGTGTGACATACG	CCGCTCGAGCCTTTAGCCAGAGCCTCAGAATAAG	1512	EcoRI, XhoI	pSC2+
rnf115	CGGGATCCAAAACATGGCGGAGGCTGCT	CCGCTCGAGTCAGAAGGACCATCTCTCCG	938	BamHI, XhoI	pSC2+

Additional file 1: Table S3. Antisense RNA probes conducted for whole mount in situs hybridization

Gene	Primer	Length (bp)	vector	Linearizing	Promoter
pacrg	F: ATGAGAACCTTTGAACCTTTGGCTA<br>R:GTTGAGAAGGCAGGACTCGTAGGTGGG	696	pGEM-T easy	SalI	T7
numb	F: TGAAGACGGCGGGTAAGA<br>R: TGGTGAGGAGGCTGGTTT	860	pGEM-T easy	SacII	SP6
galnt11	F: GAGTCAAGTGCGTAAAGGC<br>R: CACATCCAAATCCGAAAA	849	pGEM-T easy	SalI	T7
tctn2	F: GTACAGAGGACCCAGAT<br>R: AAAGCATAGTTCAATCG	871	pGEM-T easy	SpeI	T7
dnah10	F: TGCGCGGTGTCAGTTTC<br>R: TGTGATTTGTGTGTGCC	1140	pGEM-T easy	SalI	T7
rnf115	F: TGTAAGGGTGAAGTGAG<br>R: GCGGTTGAGAAAAGTAG	1034	pGEM-T easy	NcoI	SP6
cfap46	F: TTGAAGGATGAAAACCC<br>R: TGAGCACAGCACAGGAC	1031	pGEM-T easy	SalI	T7
pitx2	F: TCTCCTTGCTCTCGGCT<br>R: CTTGTTCCTGGGATTCG	1288	pGEM-T easy	SpeI	T7
lefty2	F: TCAGCGTCTTGTGTTCG<br>R: TCAGTGGGGATTTGGGG	872	pGEM-T easy	SalI	T7

Additional file 1: Table S4. The frequency of each candidate CNV in normal Chinese individuals and non-heterotaxy patients with developmental delay/intellectual disability

ID	chromosome-position	Type	Size (kbp)	Frequency of NCI	Frequency of DD/ID
5	chr4:104,554,264-105,123,728	Internal dup	569.464	0	0
5	chr6:26,019,198-26,227,973	Genic dup	208.775	0	0
6	chr7:151,816,201-151,967,676	Internal dup	151.475	1	1
7	chr1:145,625,128-145,927,662	Genic del	302.534	0	0
7	chr4:48,936,820-49,093,788	Genic dup	156.968	1	1
10	chr5:115,247,380-115,683,172	Genic dup	435.792	0	0
16	chr6:54,138,106-54,277,341	Genic dup	139.235	0	0
18	chr12:173,786-356,461	Genic dup	182.675	0	0
20	chr12:123,357,010-124,310,519	Genic dup	953.509	0	0
20	chr19:47,308,130-47,418,258	Genic dup	110.128	0	0
26	chr11:60,408,411-60,465,698	Genic del	57.287	0	0
31	chr10:6,254,055-6,374,584	Internal dup	120.529	0	0
34	chr4:93,875,432-93,988,049	Genic del	112.617	0	0
39	chr8:47,398,661-48,407,568	Genic dup	1008.907	0	0
40	chr14:73,620,299-73,786,493	Genic dup	166.194	0	0
43	chr4:101,476,709-101,668,938	Genic del	192.229	0	0
59	chr2:157,170,397-157,315,649	Internal dup	145.252	0	0
59	chr6:163,549,870-163,842,358	Genic dup	292.488	0	0
59	chr9:16,826,417-16,931,236	Internal dup	104.819	0	0
63	chr3:158,198,274-158,256,949	Genic del	58.675	0	0
63	chr10:134,358,785-134,921,135	Genic dup	562.35	0	0

NCI: normal Chinese individuals; DD: developmental delay; ID: intellectual disability; Genic del: deletion of at least one coding exon; Genic dup: full duplication of at least one gene; Internal dup: duplication of internal exons

Additional file 1: Table S5. The function of the genes associated with the 19 rare CNV segments

Chromosome	Gene	OMIM number	Component	Function	Ortholog with zebrafish
1q21.1	*RNF115*	—	ring finger protein 115	**E3 ubiquitin-protein ligase**	65.56
	CD160	604463	CD160 Molecule	MHC class I receptor activity	—
	PDZK1	603831	PDZ Domain Containing 1	ion transport, second messenger cascades	56.8
	GPR89A	612821	G Protein-Coupled Receptor 89A	voltage-gated ion channel activity	90
	GPR89C	—	G Protein-Coupled Receptor 89C	voltage-gated ion channel activity	90
	PDZK1P1	—	PDZ Domain Containing 1 Pseudogene 1	Non-Protein coding RNA	—
2q24.1	*NR4A2*	601828	Nuclear Receptor Subfamily 4 Group A Member 2	RNA polymerase II regulatory region sequence-specific DNA binding	77.18
	GPD2	138430	Glycerol-3-Phosphate Dehydrogenase 2	glycerol-3-phosphate dehydrogenase activity	71.47
3q25.32	*RSRC1*	613352	Arginine And Serine Rich Coiled-Coil 1	pre-mRNA splicing	56
4q22.2	*GRID2*	602368	Glutamate Ionotropic Receptor Delta Type Subunit 2	glutamate receptor activity	72.36
4q24	*EMCN-IT3*	—	Long Intergenic Non-Protein Coding RNA	Non-Protein coding RNA	—
4q24	*TACR3*	162332	Tachykinin Receptor 3	G-protein coupled receptor activity	66.05
5q23.1	*AP3S1*	601507	Adaptor Related Protein Complex 3 Sigma 1 Subunit	transporter activity	—
	AQPEP	—	laeverin	peptidase activity	31
	LOC644100	—	ADP ribosylation factor like GTPase 14 effector protein like	—	—
	COMMD10	616704	COMM Domain Containing 10	protein binding	60.1
6p12.1	*TINAG*	606749	Tubulointerstitial Nephritis Antigen	adhesion of proximal tubule epithelial cells	—
6p22.2	*HIST1H3A*	602810	Histone Cluster 1 H3 Family Member A	DNA binding	82.11
	HIST1H4A	602822	Histone Cluster 1 H4 Family Member A	DNA binding	80.26
	HIST1H4B	602829	Histone Cluster 1 H4 Family Member B	DNA binding	80.91
	HIST1H3B	602819	Histone Cluster 1 H3 Family Member B	DNA binding	—
	HIST1H2AB	602795	Histone Cluster 1 H2A Family Member B	DNA binding	—
	HIST1H2BB	602803	Histone Cluster 1 H2B Family Member B	DNA binding	—
	HIST1H3C	602812	Histone Cluster 1 H3 Family Member C	DNA binding	80.88

	HIST1H1C	142710	Histone Cluster 1 H1 Family Member C	DNA binding	72
	HFE	613609	Hemochromatosis	antigen binding	—
	HIST1H4C	602827	Histone Cluster 1 H4 Family Member C	DNA binding	100
	HIST1H1T	142712	Histone Cluster 1 H1 Family Member T	DNA binding	—
	HIST1H2BC	602847	Histone Cluster 1 H2B Family Member C	DNA binding	—
	HIST1H2AC	602794	Histone Cluster 1 H2A Family Member C	DNA binding	—
	HIST1H1E	142220	Histone Cluster 1 H1 Family Member E	DNA binding	74
	HIST1H2BD	602799	Histone Cluster 1 H2B Family Member D	DNA binding	—
	HIST1H2BE	602805	Histone Cluster 1 H2B Family Member E	DNA binding	—
	HIST1H4D	602823	Histone Cluster 1 H4 Family Member D	DNA binding	77.67
	HIST1H3D	602811	Histone Cluster 1 H3 Family Member D	DNA binding	84.31
	HIST1H2AD	602792	Histone Cluster 1 H2A Family Member D	DNA binding	—
	HIST1H2BF	602804	Histone Cluster 1 H2B Family Member F	DNA binding	—
	HIST1H4E	602830	Histone Cluster 1 H4 Family Member E	DNA binding	83.17
	HIST1H2BG	602798	Histone Cluster 1 H2B Family Member G	DNA binding	—
	HIST1H2AE	602786	Histone Cluster 1 H2A Family Member E	DNA binding	—
	HIST1H3E	602813	Histone Cluster 1 H3 Family Member E	DNA binding	83.58
6q26	*PACRG*	608427	Parkin Coregulated	**correlated with motile cilia during development; ubiquitin protein ligase binding**	91
	PACRG-AS1	—	PACRG Antisense RNA 1	Non-Protein coding RNA	—
	DKFZp451B082	—	Uncharacterized LOC401282	Non-Protein coding RNA	—
	CAHM	615930	Colon Adenocarcinoma Hypermethylated	Non-Protein coding RNA	—
	QKI	609590	QKI, KH Domain Containing RNA Binding	RNA binding, myelinization	80.3
8q11.1q11.21	*LINC00293*	—	Long Intergenic Non-Protein Coding RNA 293	Non-Protein coding RNA	—
	LOC100287846	—	Patched 1 Pseudogene	Pseudogene	—
	KIAA0146	615384	KIAA0146 ortholog/Scaffolding Protein Involved In DNA Repair	DNA double-strand break (DBS) repair	49.13
9p22.2	*BNC2*	608669	Basonuclin 2	zinc ion binding	61

10p15.1	*PFKFB3*	605319	6-Phosphofructo-2-Kinase/Fructose-2,6-Biphosphatase 3	6-phosphofructo-2-kinase activity	71.41
	LOC399715	—	Uncharacterized LOC399715	Non-Protein coding RNA	—
10q26.3	*INPP5A*	600106	Inositol Polyphosphate-5-Phosphatase A	inositol-polyphosphate 5-phosphatase activity	74
	NKX6-2	605955	NK6 Homeobox 2	DNA binding, transcription factor activity	72.99
	TTC40	—	Cilia And Flagella Associated Protein 46	**cilium movement**	52.04
	LOC399829	—	long intergenic non-protein coding RNA 1168	Non-Protein coding RNA	—
	GPR123	612302	G-Protein Coupled Receptor 123	transmembrane signaling receptor activity	62.25
11q12.2	*LINC00301*	—	Long Intergenic Non-Protein Coding RNA 301	Non-Protein coding RNA	—
12p13.33	*IQSEC3*	612118	IQ Motif And Sec7 Domain 3	ARF guanyl-nucleotide exchange factor activity	62
	LOC574538	—	Uncharacterized LOC574538	RNA Gene	—
	SLC6A12	603080	Solute Carrier Family 6 Member 12	neurotransmitter transporter	62
	SLC6A13	615097	Solute Carrier Family 6 Member 13	Sodium-dependent GABA and taurine transporter	74
12q24.31	*VPS37B*	610037	acuolar Protein Sorting 37 Homolog B	a regulator of vesicular trafficking process	60.4
	ABCB9	605453	ATP Binding Cassette Subfamily B Member 9	ATP binding	59.58
	OGFOD2	—	2-Oxoglutarate And Iron Dependent Oxygenase Domain Containing 2	oxidoreductase activity	58.36
	ARL6IP4	607668	ADP Ribosylation Factor Like GTPase 6 Interacting Protein 4	modulates alternative pre-mRNA splicing	44
	PITPNM2	608920	Phosphatidylinositol Transfer Protein Membrane Associated 2	phosphatidylinositol transporter activity	56
	MIR4304	—	MicroRNA 4304	RNA Gene	—
	LOC100507091	—	PITPNM2 antisense RNA 1	Non-Protein coding RNA	—
	MPHOSPH9	605501	M-Phase Phosphoprotein 9	M-phase enzymatic activities	38
	C12orf65	613541	Chromosome 12 Open Reading Frame 65	translation release factor	56
	CDK2AP1	602198	Cyclin Dependent Kinase 2 Associated Protein 1	specific inhibitor of the cell-cycle kinase CDK2	71.65
	SBNO1	614274	Strawberry Notch Homolog 1	molecular function	70.25
	SETD8	607240	SET Domain Containing (Lysine Methyltransferase) 8	methyltransferase activity	65.26

	RILPL2	614093	Rab Interacting Lysosomal Protein Like 2	cellular protein transport	61.46
	SNRNP35	—	Small Nuclear Ribonucleoprotein U11/U12 Subunit 35	RNA binding	69.25
	RILPL1	614092	Rab Interacting Lysosomal Protein Like 1	cellular protein transport	71.17
	MIR3908	—	MicroRNA 3908	RNA Gene	—
	TMED2	—	Transmembrane P24 Trafficking Protein 2	vesicular protein trafficking	80.15
	DDX55	—	DEAD-Box Helicase 55	ATP-binding RNA helicase	69.03
	EIF2B1	606686	Eukaryotic Translation Initiation Factor 2B Subunit Alpha	translation initiation factor	77
	GTF2H3	601750	General Transcription Factor IIH Subunit 3	protein kinase activity, damaged DNA binding	71
	TCTN2	613846	Tectonic Family Member 2	**hedgehog signaling transduction, regulate ciliogenesis**	48.17
	ATP6V0A2	611716	ATPase H+ Transporting V0 Subunit A2	ATPase binding	67
	DNAH10	605884	Dynein Axonemal Heavy Chain 10	**microtubule motor activity, ATPase activity**	67.65
14q24.2	*PSEN1*	104311	Presenilin 1	endopeptidase activity	70.82
	PAPLN	—	Papilin, Proteoglycan Like Sulfated Glycoprotein	metalloendopeptidase activity	54.68
	NUMB	603728	NUMB, Endocytic Adaptor Protein	**suppress notch activity, protein binding**	65.28
19q13.32	*SNAR-E*	—	Small ILF3/NF90-Associated RNA E	RNA Gene	—
	AP2S1	602242	Adaptor Related Protein Complex 2 Sigma 1 Subunit	contributes to clathrin adaptor activity	85.68

Additional file 1: Table S6. The bioinformatics information on the variant of *LEFTY1* in the patient with CNV of *TTC40* (*CFAP46*)

ID	Gene	Mutation site	Amino acid change	Exonic Function	SIFT score	SIFT pred	PP2 HDIV score	PP2 HDIV pred	Mutation Taster score	Mutation Taster pred
63	*LEFTY1*	c. 841 A > G	p.Trp281Arg	nonsynonymous	0	Del	0.957	Dam	1	Dis

PP2: Polyphen2; pred: predicting; Del: Deleterious; Dam: Probably damaging; Dis: disease_causing

Additional file 1: Table S7. The bioinformatics information on the variants of candidate genes

ID	Gene	Mutation site	Amino acid change	Exonic Function	SIFT score	SIFT pred	PP2 HDIV score	PP2 HDIV pred	Mutation Taster score	Mutation Taster pred
10	*DNAH10*	c.G11888A	p.R3963H	nonsynonymous	0	Del	1	Dam	1	Dis
21	*DNAH10*	c.T12026C	p.L4009P	nonsynonymous	0	Del	1	Dam	1	Dis
79	*DNAH10*	c.C10075T	p.L3359F	nonsynonymous	0	Del	1	Dam	1	Dis
93	*RNF115*	c.G799A	p.V267I	nonsynonymous	0.16	Tol	0.1	Ben	1	Dis
5	*TCTN2*	c.C91T	p.P31S	nonsynonymous	0	Del	1	Dam	0.999	Dis
101	*NUMB*	c.C518T	p.A173V	nonsynonymous	0.12	Tol	1	Dam	1	Dis

PP2: Polyphen2; pred: predicting; Del: Deleterious; Tol: tolerated; Dam: Probably damaging; Ben: benign; Dis: disease_causing
