# Supplementary material for: Rare copy number variants analysis identifies novel candidate genes in heterotaxy syndrome patients with congenital heart defects
Source: Genome Med. 2018 May 30;10:40. doi: 10.1186/s13073-018-0549-y (PMC5975672; doi:10.1186/s13073-018-0549-y)
Supplement: Supplementary file 2 — Figure S1. Knockdown efficiency of splice blocking and translation blocking MOs in zebrafish embryos. Figure S2. The knockout efficiency of dnah10 and rnf115 by CRISPR/Cas9. Figure S3. Chromosomal view of rare CNVs in candidate Htx patients and the verified results of qPCR. Figure S4. Gene sequencing peak shows a nonsynonymous heterozygous mutation in LEFTY1 in the patient with CNV of TTC40 (CFAP46). Figure S5. Rare variations were detected in Htx patients. (PDF 792 kb) [file 13073_2018_549_MOESM2_ESM.pdf]

Additional file 2：Supplemental Figures S1-S5

Additional file 2: Fig. S1. Knock-down efficiency of splice blocking and translation blocking MOs in zebrafish embryos.

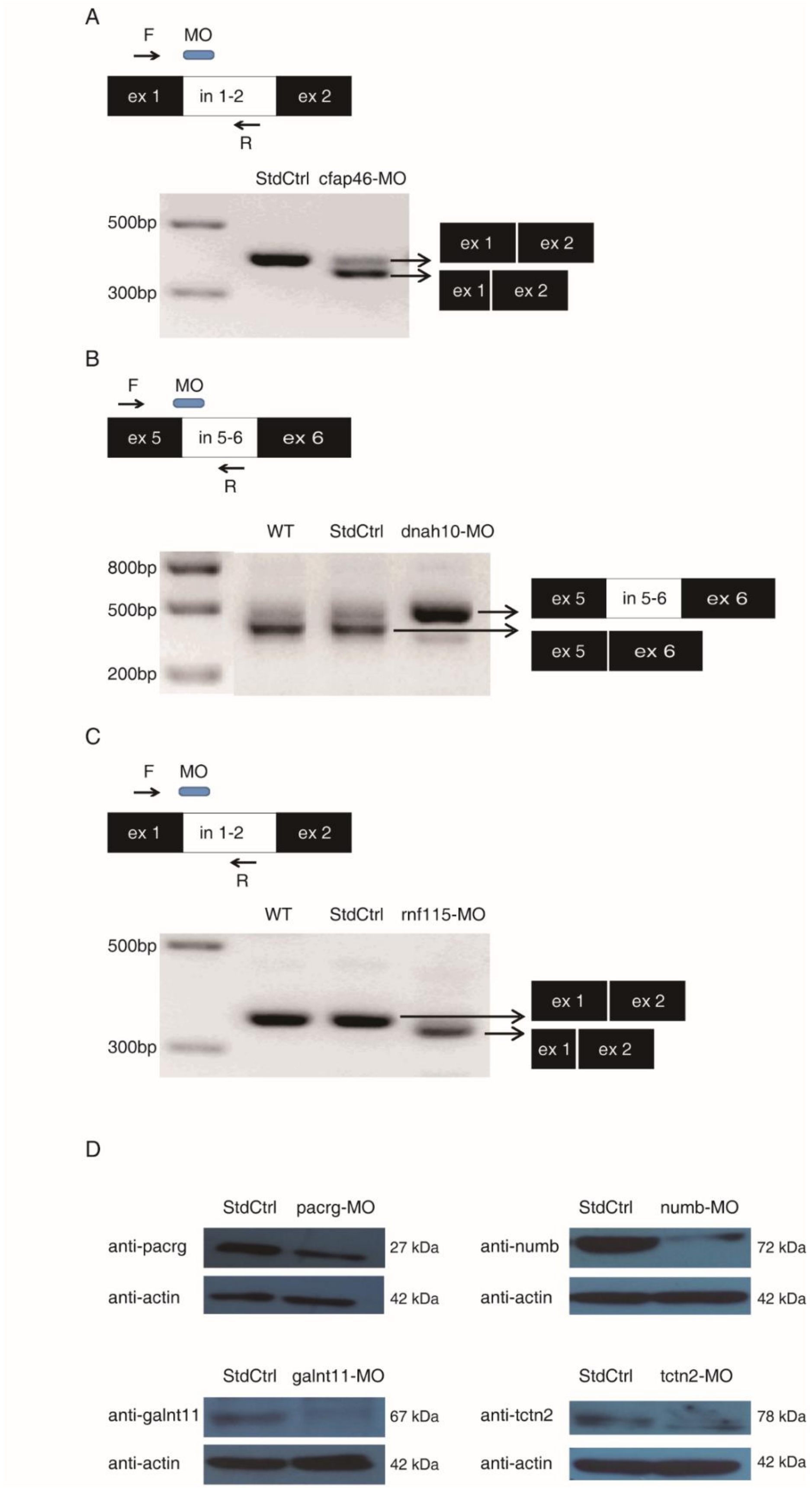

(A) The *cfap46* splice blocking morpholino (sb-MO) targets the junction of exon 1 and intron 1-2, which results in a shorter exon 1 might because of a cryptic splice site activation. (B) The *dnah10* sb-MO targeting the junction of exon 5 and intron 5-6 can cause intron 5-6 insertion. (C) The *rnf115* sb-MO targeting the junction of exon 1 and intron 1-2 results in a shorter exon 1 might because of a cryptic site activation. (D) Use western blot to verify the efficiency of MOs targeting *numb*, *galnt11*, *pacrg* and *tctn2*. The results show knockdown of protein expression in relevant morphants. Anti-actin was used as a loading control.

In A-C, RT-PCR (reverse transcription-PCR) were conducted to analyze the efficiency of sb-MOs targeting *cfap46*, *dnah10* and *rnf115*. Total RNA were extracted from 2 dpf wide-type zebrafish embryos. (Upper) A schematic illustrates the target locations of MO and RT-PCR primers. (Lower) Agarose electrophoresis and Sanger sequencing results of PCR amplification products. StdCtrl, standard control; ex, exon; in, intron; WT, wide type.

Additional file 2: Fig. S2. The knockout efficiency of *dnah10* and *rnf115* by CRISPR/Cas9.

The knockout efficiency of dnah10：11/13, 84.6%

ATGGCTCAGTTCTATGCTTACTGGGAGCGCAAGA WT (42#,47#)
ATGGCTCAGTTCTATGCT------ GGGAGCGCAAGA -4bp（41#，44#，46#，414#）
ATGGCTCAGTTCTATGCTTCTATGCTAGTACTGGGAGCGCAAGA +10bp （43#）
ATGGCTCAGTTCTATGC--------------------GCAAGA -11bp （45#）
ATGGCTCAGTTCTATGCTT-----GGGAGCGCAAGA -3bp （48#）
ATGGCTCAGTTCTATGCTTTTTGATCAGTTCTATGCTGA -13bp+18（410#）
ATGGCTCAGTTCTATGCTGAGCGGGAGCGCAAGA -4bp+4bp（411#，412#）
ATGGCTCAGTCA------------------ GGGAGCGCAAGA -12bp+2 （415#）

The knockout efficiency of rnf115：8/12, 75%

ATCTGGACAGTCTTGACTCTGAGCGGCTTCCAGGAGGGG WT（43#,46#,47#,415#）
ATCTGGACAGTCTTGACTTCCAGCGGCTTCCAGGAGGGG -3+3bp (41#)
ATCTGGACAGTCTTGA-----------GCGGCTTCCAGGAGGGG -6bp (44#)
ATCTGGACAGTCTTGACTC------- --GGCTTCCAGGAGGGG -5bp (45#)
ATCTGGAC------------------ --------GCGGCTTCCAGGAGGGG -14bp (48#)
ATCTGGACAGTCTTGACT--TGAGCGGCTTCCAGGAGGGG -1bp (411#)
ATCTGGACAGTCTTGA-----------GCGGCTTCCAGGAGGGG -6bp (412#)
ATCTGGACAGTCTTGACTCCAGTCTTGAGCGGCTTCCAGGAGGGG +6bp (413#)
ATCTGGACAGTCTTGACTCTTGAGCGGCTTCCAGGAGGGG +1bp (414#)

(A) Sequence analysis of *dnah10* mutations caused by co-injection of zebrafish codon-optimized Cas9 mRNA and *dnah10* gRNA. The red fonts show the target site of *dnah10* gRNA, yellow blanks show mutated sequences, and the green fonts show the PAM sequence.
(B) Sequence analysis of *rnf115* mutations caused by co-injection of zebrafish codon-optimized Cas9 mRNA and *rnf115* gRNA. The red fonts show the target site of *rnf115* gRNA, yellow blanks show mutated sequences, and the green fonts show the PAM sequence.

Additional file 2: Fig. S3. Chromosomal view of rare CNVs in candidate Htx patients and the verified results of qPCR.

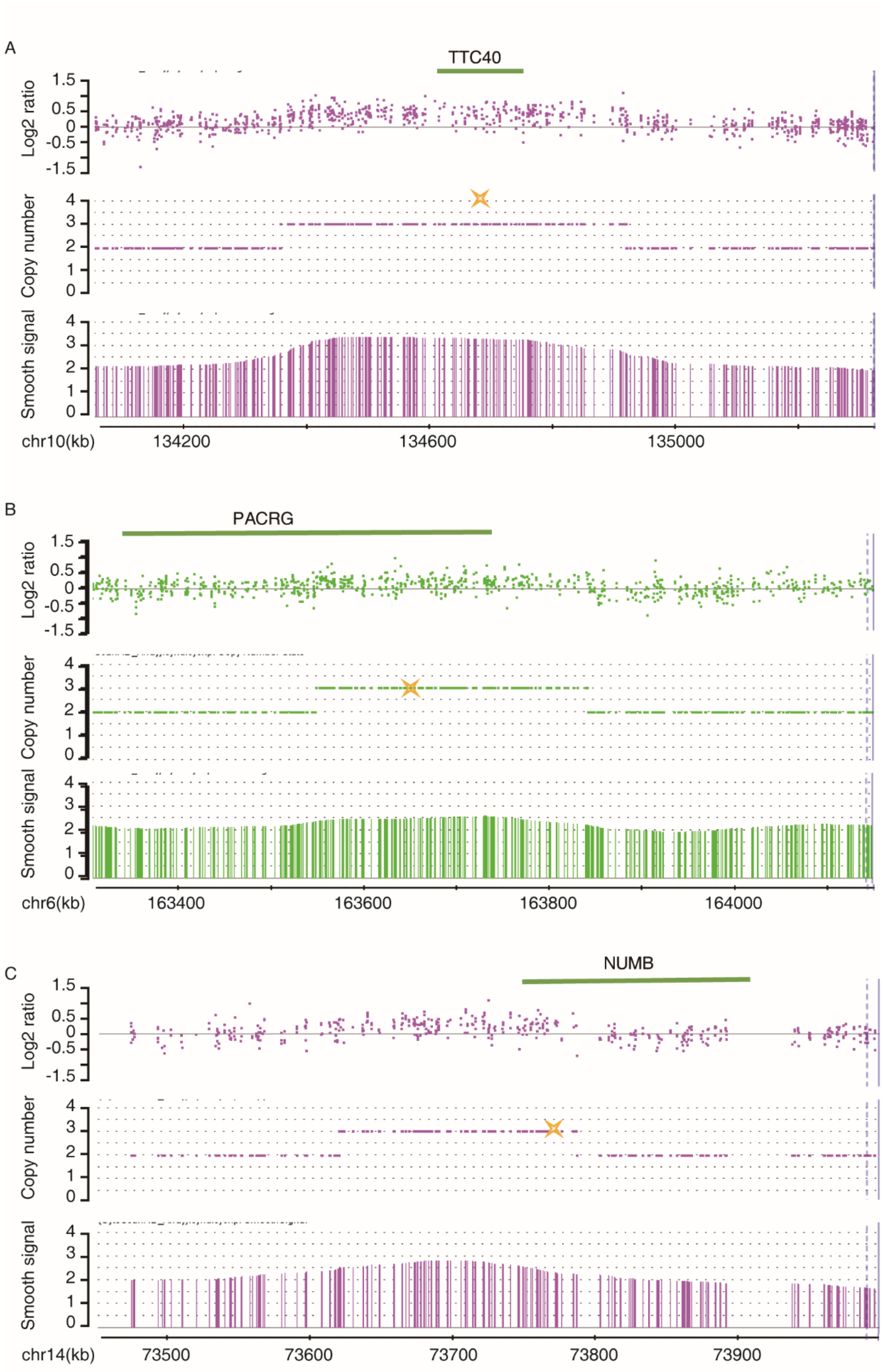

(A) A 562 kb duplication at 10q26.3 encompassing *TTC40* (*CFAP46*). (B) A 292.5 kb duplication at 6q26 encompassing *PACRG*. (C) A 166 kb duplication of 14q24.2 involving *NUMB*.

The upper panel depicts Log2 ratio data, the middle panel depicts the copy number duplications or deletions, and the lower panel depicts smooth signals of indicated segments. Locations of genes implicated in Htx are shown in the top. Results of qPCR are denoted by yellow stars.

Additional file 2: Fig. S4. Gene sequencing peak shows a nonsynonymous heterozygous mutation in *LEFTY1* in the patient with CNV of *TTC40* (*CFAP46*).

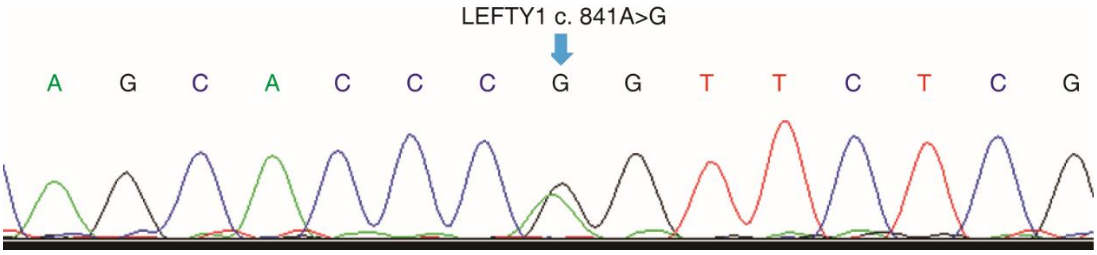

The arrow indicates the altered nucleotides of c. 841 A>G in *LEFTY1*.

Additional file 2: Fig. S5. Rare variations were detected in Htx patients.

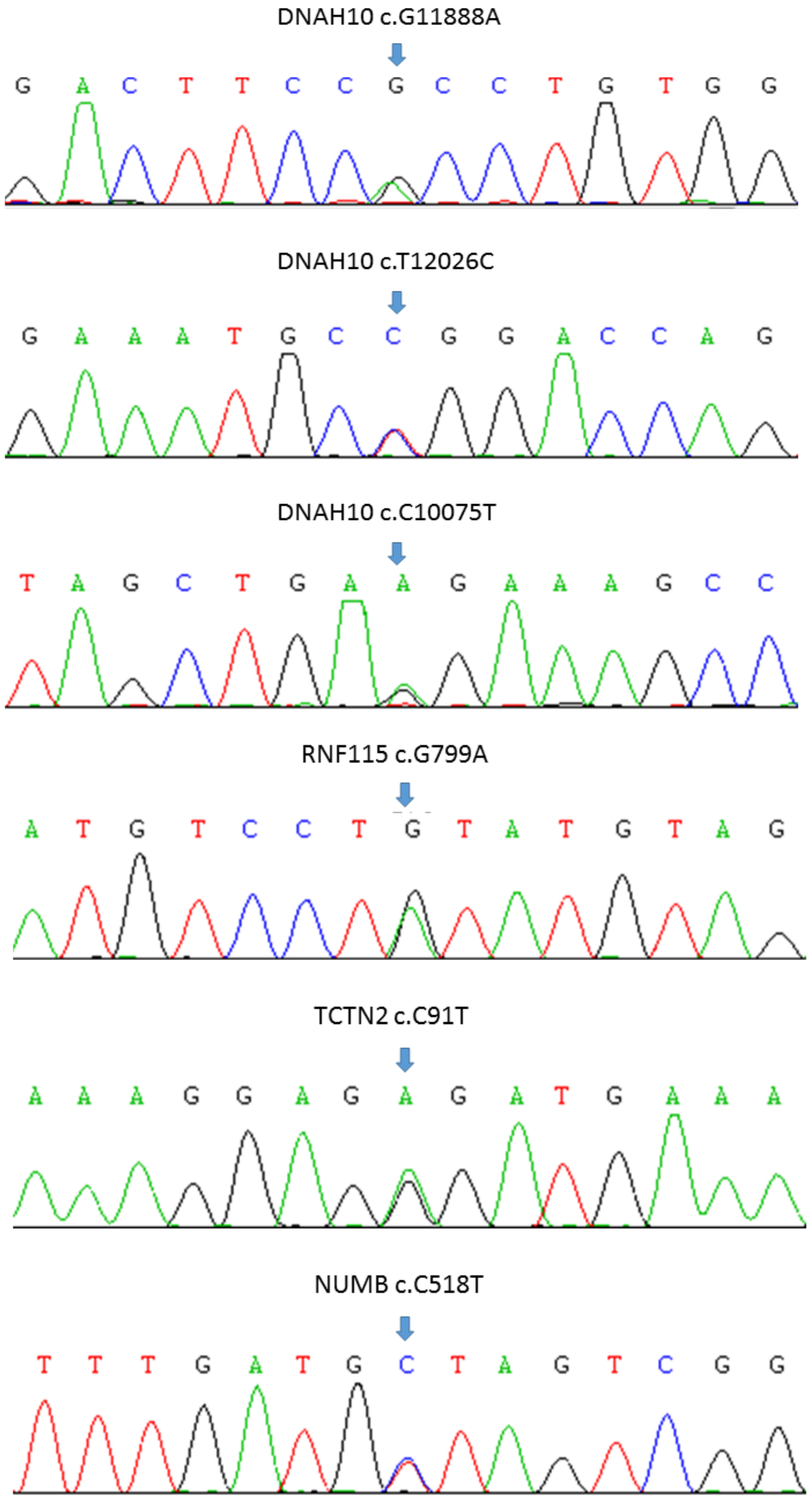

Rare heterozygous mutations in candidate genes *DNAH10* (c.G11888A/ p.R3963H; c.T12026C/ p.L4009P; c.C10075T/ p.L3359F), *RNF115* (c.G799A/ p.V267I), *TCTN2* (c.C91T/ p.P31S), and *NUMB* (c.C518T/ p.A173V) were detected in six sporadic Htx patients.
The arrow indicates the altered nucleotides.

Additional material

Knock-down efficiency of splice blocking and translation blocking MOs in zebrafish embryos.

To analyze the efficiency of splice-blocking and ATG-blocking MOs, we injected 8 ng *pacrg* MO, 8 ng *tctn2* MO, 8 ng *numb* MO, 8 ng *dnah10* MO, 2 ng *rnf115* MO, 8 ng *cfap46*/*ttc40* MO, 8 ng *galnt11* MO per embryo, then conducted RT-PCR (reverse transcription PCR) and Western blot experiments, the results of which are demonstrated in **Additional file 2: Fig. S1**.
